# Induction Chemotherapy Followed by either Chemoradiotherapy or Bioradiotherapy in Laryngeal Cancer

**DOI:** 10.31557/APJCP.2021.22.5.1633

**Published:** 2021-05

**Authors:** Mohammad Hasan Larizadeh, Fatemeh Mohammadi, Mohammad Shabani, Mohammad Ali Damghani

**Affiliations:** 1 *Neuroscience Research Center, Neuropharmacology Institute, Kerman University of Medical Sciences, Kerman, Iran. *; 2 *Department of Ear, Nose and Throat, Shafa Hospital, Kerman University of Medical Sciences, Kerman, Iran. *

**Keywords:** Laryngeal cancer, cetuximab, chemotherapy, radiotherapy

## Abstract

**Objective:**

In loco regionally advanced head and neck cancer, the superiority of concomitant cetuximab with radiation over radiation alone has been proven previously. But comparison between chemo radiation and bioradiation has not been well studied.

**Methods:**

Between October 2013 and August 2017, 38 patients with locoregionally advanced laryngeal cancer and more than 50% response to 3 cycles of induction chemotherapy (docetaxel and cisplatin: both with a dose of 75 mg/m^2^ on the first day and 5-flurouracil: 750 mg/m^2^ during days 1to 3; repeated every 21 days) were selected to receive either carboplatin (18 patients, AUC 1.5 , weekly) or cetuximab (20 patients, with loading dose of 400 mg/m^2^ and weekly dose of 250 mg/m2) with radiation. A Kaplan–Meier analysis was used to calculate progression free survival and overall survival rates. The log–rank test was used to compare overall survival between treatment groups.

**Results:**

The median follow up time was 36 months. The 2-year organ preservation rate of 78.9% was achieved. The 3- year progression-free survival rates of 65.2%, 72.7% and 58.2% were observed for all patients, carboplatin group and cetuximab group, respectively (p=0.4). The 3-year estimates of overall survival were 67.8%, 69.2 %, and 66.3 % for all patients, carboplatin group and cetuximab group, respectively (p=0.47). Concomitant carboplatin was discontinued in 3 patients due to toxicity

**Conclusion:**

Concomitant cetuximab is a reasonable alternative to concomitant chemotherapy. But the difference in treatment outcome between bioradiation and chemoradiation remains to be defined.

## Introduction

Treatment outcome for locally advanced squamous cell carcinoma of head and neck (HN¬SCC) is very poor (Larizadeh and Shabani, 2012; Shabani and Larizadeh, 2015). Recently, a trend has been made for adding cetuximab to the multimodality protocols to improve treatment outcome and to reduce chemotherapy related toxicity (Mehra et al., 2008; Sharafinski et al., 2010; Agulnik, 2012). Cetuximab is an IgG1 monoclonal antibody and inhibits epithelial growth factor receptor (EGFR) (Agulnik, 2012; Sacco and Worden, 2016). Treatment outcome has been improved with the addition of cetuximab to radiation(Bonner et al., 2010). However, there is no phase III trial to compare concomitant chemotherapy versus concomitant cetuximab with radiation. A few retrospective studies have been conducted to compare chemoradiation with bioradiation (Koutcher et al., 2011; Ley et al., 2013; Levy et al., 2014; Shapiro et al., 2014; Strom et al., 2015; Tang et al., 2015; Riaz et al., 2016). Only one phase II trial was conducted to compare chemoradiation versus bioradiation in sequential modality(Lefebvre et al., 2013). According to our knowledge this is the second study in which cetuximab has been compared with chemotherapy during concomitant phase of sequential approach. We are to compare the survival outcome of induction chemotherapy followed by either chemoradiation or bioradiation. Another purpose is to define laryngeal preservation rate with cetuximab that has been rarely reported, previously. 

## Materials and Method

Between October 2013 and August 2017, 38 patients with T3, T4 or N+ laryngeal cancer were selected to receive induction chemotherapy followed by either bioradiotherapy or chemoradiotherapy. Beside a laryngoscopy study, computed tomography (CT) scans of the neck and chest X-ray were used for staging. Other imaging studies were performed, whenever clinically indicated. Treatment response was assessed by indirect laryngoscopy or CT scan (when indicated). The 1988 American Joint Committee for Cancer staging system was used for staging. Exclusion criteria included fewer than 50% clinical response to induction chemotherapy, presence of a second primary tumor or distant metastasis, abnormal hematological, renal and liver function and Eastern Cooperating Oncology Group performance status of 2. No matching was done to select between 2 protocols. Induction chemotherapy consisted of three cycles of docetaxel (75 mg/m^2^ on first day), cisplatin (75 mg/m^2^ on first day) and 5-flurouracil (5-FU) (750 mg/m^2^, from days 1to 3) (TPF). Responder patients to induction chemotherapy were selected to receive 3 – Dimensional conformal radiotherapy. It was started 4–6 weeks after the last cycle of chemotherapy. A total dose of 70 Gy and50 GY was given to the gross tumors and subclinical diseases, respectively. The field size was reduced after 45 Gy to spare the spinal cord. During radiotherapy phase, carboplatin (AUC: 1.5) was given weekly. Cetuximab with a loading dose of 400mg/ m^2^ and weekly dose of 250mg/m^2^ was used. Those patients with no complete response to chemoradiation or with local recurrence underwent laryngectomy, whenever it was possible. A Kaplan–Meier analysis was used to calculate survival outcomes. The log–rank test was used to compare overall survival between treatment groups. The time between the first dates to the last dates of visit was used to calculate overall survival. The time between the first dates of visit to recurrence dates was used to calculate progression free survival. The laryngeal preservation rate was defined as freedom from either local recurrence or from the need for salvage surgery at the primary site. No need for surgery and no evidence for recurrence were necessary for calculation of laryngeal preservation rate. The National Cancer Institute Common Toxicity Criteria (version 2) was used for toxicity grading. 

## Results

Table 1 showes the patient characteristics. During follow up periods (median: 36 months, range: 12 to 72 months), disease progression was seen in 11 patients (28.9%). Loco regional and distant failure was occurred in 9 and 1 patients, respectively. Failure in multiple sites was seen in one patient. Laryngectomy was performed in 6 patients due to local recurrence or no complete response to chemoradiation. Three patients underwent neck dissection (one for N3 disease, one for persistent and one for recurrent nodal disease). 

**Figure 1:**
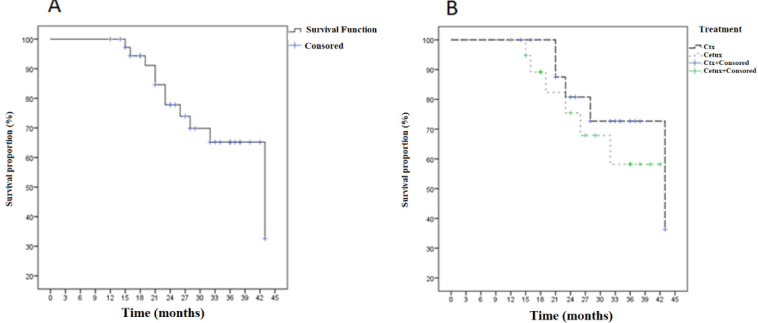
Kaplan–Meier Progression Free Survival Curve for All Patient (A) and Treatment Groups (B).

The 2-year organ preservation rate was 78.9%. The 2 and 3years progression-free survival rates of 77.8 and 65.2% were achieved, respectively. The difference in progression free survival according to treatments was not significant (p=0.4). For carboplatin group the 2 and 3years progression-free survival rates of 80 and 72.7% were observed, respectively. For cetuximab group the 2 and 3years progression-free survival rates of 75.4 and 58.2% were achieved, respectively (Figure 1).

**Figure 2:**
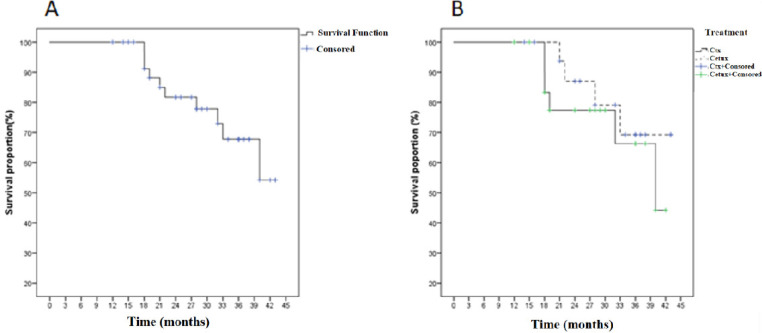
Kaplan–Meier Overall Survival Curve for All Patients(A) and Treatment Groups (B).

For all patients overall survival rates after 2 and 3 years were 81.7 and 67.8 %, respectively. Two-year and 3-year estimates of overall survival were 87.1 and 69.2 %, respectively, for carboplatin group and 77.4 and 66.3 % for cetuximab group (P = 0.47) (Figure 2). 

High-grade adverse events are shown in Table 2. During induction chemotherapy, hematologic events were the most frequent toxicity. Chemotherapy was postponed in eight patients during induction phase. The most frequent toxicity during concomitant phase was mucositis. In cetuximab group, there was no need to terminate planned treatment due to toxicity. Concomitant carboplatin was discontinued in 3 patients due to adverse events. No death was seen from toxic effects.

**Table 1:** The Patient Characteristics According to Treatment

Characteristic	Carboplatin group 18 patients	Cetuximab group 20 patients
Age		
Median(year)	54	57
Range	42 to71	43 to 68
Sex		
Male	16 (88.8%)	17 (85%)
Female	2 (11.2%)	3 (15%)
Site		
Supraglottal	14 (77.8%)	16 (80%)
Gluttal	4 (22.2%)	4 (20%)
N stage		
N0	12 (66.6%)	15 (75%)
N1-3	6 (33.4)	5 (25%)
Tumor stage		
T2	2 (11.2%)	1 (5%)
T3-4	16 (88.8%)	19 (95%)

**Table 2:** High Grade Toxicity According to Treatment

High grade Toxicity	Induction chemotherapy Number (%)	Concomitant Carboplatin Number (%)	Concomitant Cetuximab Number (%)
Mucositis	4 (10.5%)	8 (44.4%)	7 (35%)
Skin	0	1 (5.5%)	9 (45%)
Hematologic	17 (44.7%)	3 (16.6%)	0
Renal	2 (5.2)	0	0

## Discussion

Recently, incorporation of cetuximab to induction and/ or concomitant phase of chemoradiation has been an area of interest (Larizadeh, 2017). The superiority of concomitant cetuximab and radiotherapy compared with radiation alone has been proven with a phase III study (Bonner et al., 2010). But there is no phase III trial to compare chemoradiation with concomitant cetuximab and radiation (Larizadeh, 2017). A phase II trial compared radiotherapy with concomitant cisplatin versus concomitant cetuximab. Loco regional control and survivals were similar between the treatment arms (Magrini et al., 2016). 

Also, the addition of cetuximab to induction or concomitant phase of sequential modalities has been studied. A phase II trial conducted by Kies et al., (2010) to evaluate combination of cetuximab with chemotherapy for induction treatment followed by local therapy in HNSCC. Achieving the 3- overall survival rate of 91%, they concluded that induction therapy with cetuximab / paclitaxel and carboplatin followed local therapy is an effective treatment with tolerable toxicity profile (Kies et al., 2010).

In another phase II study conducted by Mesia et al., (2016) unresectable HNSCC were selected to receive induction therapy consisted of four 21-day cycles of TPF and cetuximab, followed by radiation and weekly cetuximab. The 2year locoregional control rate was 57%. The median overall survival was 40.7 months (Mesía et al., 2016).

A phase II ECOG- ACRIN trial was designed to evaluate the treatment results of induction cetuximab, paclitaxel, and carboplatin followed by chemoradiation for locally advanced HNSCC. Weekly cetuximab, paclitaxel and carboplatin were administered throughout radiation. Overall survival was 78% at 3 years. They concluded that sequentional modality containing cetuximab is safe with high response rate and promising survival(Wanebo et al., 2014).

Another phase II study was done by Argiris et.al (2010) Locally advanced HNSCC were treated with 3 cycles of docetaxel, cisplatin and weekly cetuximab. After induction chemotherapy, weekly cisplatin and cetuximab was given during radiation. It was followed by 6 months cetuximab therapy as maintenance .The 3- year progression-free survival and overall survival rates of 70% and 74% was observed, respectively (Argiris et al., 2010).

In all of these studies cetuximab has been used during induction phase of sequential modalities. Only one randomized phase II Study (TREMPLIN study) was designed to compare the treatment outcomes of induction chemotherapy followed by chemoradiotherapy or bioradiotherapy. After induction chemotherapy with TPF, the patients were randomly assigned to receive concomitant cisplatin (100 mg/m^2^ every 3 weeks, started on day 1) or cetuximab (400 mg/m^2^as a loading dose followed by 250 mg/m^2^, weekly) with radiation.

The 3-month larynx preservation rate of 95% and 93% was seen for cisplatin and cetuximab group, respectively. Overall survival between two groups did not differ. It was 75% for cisplatin and 73% for cetuximab after a median follow-up of 36 months (Lefebvre et al., 2013). Like TREMPLIN study overall survival between treatment arms had no difference in our study. But one limitation of our study is low number of the patients. Unlike TREMPLIN study, we used carboplatin instead of cisplatin during concomitant phase of treatment. This protocol was similar to TAX 323 study.

In TREMPLIN, receiving full protocol was achieved in 43 % and 77% of patient with chemotherapy and biotherapy arm, respectively. Receiving full protocol in our study was achieved in 83% and 100% of patients with chemoradiation and bioradiation, respectively. It seems that weekly carboplatin can be tolerated more than high dose cisplatin and its treatment outcome is similar to cetuximab. The superiority of cetuximab over low toxic carboplatin base regimens is not clear.

In previous our study, TPF induction chemotherapy followed by radiation alone showed the 2 and 3 years overall survival rate and the 2-year laryngeal preservation rate of 83%, 71% and 75%, respectively (Larizadeh and Damghani, 2010). This new study of induction chemotherapy followed by concomitant radiation showed the 2 and 3years overall survival rate and the 2-year laryngeal preservation rate of 81.7%, 67.8% and 78.9%, respectively. The role of concomitant chemotherapy or biotherapy for partial or complete responders to induction chemotherapy is unclear (Larizadeh, 2017).

In conclusion according to our study bioradiation can be a reasonable alternative for chemoradiation in sequential modalities, although one limitation for this study is low number of patients. What is to be defined is that after induction chemotherapy which patients should be considered for concomitant treatment. Also, the selection between chemoradiation and bioradiation remains to be defined.

## Author Contribution Statement

MHL and MAD conceived and designed the concept and road map of the study, searched the literature collected the data, and drafted the manuscript. FM and MS have critically reviewed the manuscript, designed the study, and helped in manuscript preparation and revision. All authors have made substantive contribution and attest to approving the final manuscript.

## References

[B1] Agulnik M (2012). New approaches to EGFR inhibition for locally advanced or metastatic squamous cell carcinoma of the head and neck (SCCHN). Med Oncol.

[B2] Argiris A, Heron DE, Smith RP (2010). Induction docetaxel, cisplatin, and cetuximab followed by concurrent radiotherapy, cisplatin, and cetuximab and maintenance cetuximab in patients with locally advanced head and neck cancer. J Clin Oncol.

[B3] Bonner JA, Harari PM, Giralt J (2010). Radiotherapy plus cetuximab for locoregionally advanced head and neck cancer: 5-year survival data from a phase 3 randomised trial, and relation between cetuximab-induced rash and survival. Lancet Oncol.

[B4] Kies MS, Holsinger FC, Lee JJ (2010). Induction chemotherapy and cetuximab for locally advanced squamous cell carcinoma of the head and neck: results from a phase II prospective trial. J Clin Oncol.

[B5] Koutcher L, Sherman E, Fury M (2011). Concurrent cisplatin and radiation versus cetuximab and radiation for locally advanced head-and-neck cancer. Int J Radiat Oncol Biol Phys.

[B6] Larizadeh MH (2017). Cetuximab for squamous cell carcinoma of the head and neck. Int J Cancer Manage.

[B7] Larizadeh MH, Damghani MA (2010). Sequential chemoradiotherapy in advanced laryngeal cancer: an institutional experience. Asia Pac J Clin Oncol.

[B8] Larizadeh MH, Shabani M (2012). Survival following non surgical treatments for oral cancer: a single institutional result. Asian Pac J Cancer Prev.

[B9] Lefebvre JL, Pointreau Y, Rolland F (2013). Induction chemotherapy followed by either chemoradiotherapy or bioradiotherapy for larynx preservation: the TREMPLIN randomized phase II study. J Clin Oncol.

[B10] Levy A, Blanchard P, Bellefqih S (2014). Concurrent use of cisplatin or cetuximab with definitive radiotherapy for locally advanced head and neck squamous cell carcinomas. Strahlentherapie und Onkologie.

[B11] Ley J, Mehan P, Wildes TM (2013). Cisplatin versus cetuximab given concurrently with definitive radiation therapy for locally advanced head and neck squamous cell carcinoma. Oncology.

[B12] Magrini SM, Buglione M, Corvò R (2016). Cetuximab and radiotherapy versus cisplatin and radiotherapy for locally advanced head and neck cancer: a randomized phase II trial. J Clin Oncol.

[B13] Mehra R, Cohen RB, Burtness BA (2008). The role of cetuximab for the treatment of squamous cell carcinoma of the head and neck. Clin Adv Hematol Oncol.

[B14] Mesía R, Vázquez S, Grau JJ (2016). A phase 2 open label, single-arm trial to evaluate the combination of cetuximab plus taxotere, cisplatin, and 5-flurouracil as an induction regimen in patients with unresectable squamous cell carcinoma of the head and neck. Int J Radiat Oncol Biol Phys.

[B15] Riaz N, Sherman E, Koutcher L (2016). Concurrent chemoradiotherapy with cisplatin versus cetuximab for squamous cell carcinoma of the head and neck. Am J Clin Oncol.

[B16] Sacco AG, Worden FP (2016). Molecularly targeted therapy for the treatment of head and neck cancer: a review of the ErbB family inhibitors. OncoTargets Ther.

[B17] Shabani M, Larizadeh MH (2015). A review of chemotherapy for locally advanced head and neck cancers. Rep Radiot Oncol.

[B18] Shapiro LQ, Sherman EJ, Riaz N (2014). Efficacy of concurrent cetuximab vs 5-fluorouracil/carboplatin or high-dose cisplatin with intensity-modulated radiation therapy (IMRT) for locally-advanced head and neck cancer (LAHNSCC). Oral Oncol.

[B19] Sharafinski ME, Ferris RL, Ferrone S (2010). Epidermal growth factor receptor targeted therapy of squamous cell carcinoma of the head and neck. Head Neck.

[B20] Strom TJ, Trotti AM, Kish J (2015). Comparison of every 3 week cisplatin or weekly cetuximab with concurrent radiotherapy for locally advanced head and neck cancer. Oral Oncol.

[B21] Tang C, Chan C, Jiang W (2015). Concurrent cetuximab versus platinum-based chemoradiation for the definitive treatment of locoregionally advanced head and neck cancer. Head Neck.

[B22] Wanebo H, Lee J, Burtness B (2014). Induction cetuximab, paclitaxel, and carboplatin followed by chemoradiation with cetuximab, paclitaxel, and carboplatin for stage III/IV head and neck squamous cancer: a phase II ECOG-ACRIN trial (E2303). Ann Oncol.

